# Supplementary material for: ZEB1, a novel junctional adhesion molecule A regulator, impacts sensitivity of pancreatic cancer-associated fibroblasts to reovirus
Source: Mol Ther Oncol. 2025 Oct 17;33(4):201071. doi: 10.1016/j.omton.2025.201071 (PMC12617759; doi:10.1016/j.omton.2025.201071)
Supplement: Document S1. Figures S1–S5 and Tables S1–S3 [file mmc1.pdf]

OMTON, Volume *33*

Supplemental information

ZEB1, a novel junctional adhesion molecule A regulator, impacts sensitivity of pancreatic cancer-associated fibroblasts to reovirus

Nicole Dam, Tom J. Harryvan, Hao Dang, Gavriil Ioannidis, Bernhard Schmierer, Lukas J. A.C. Hawinkels, and Vera Kemp

Supplemental figures

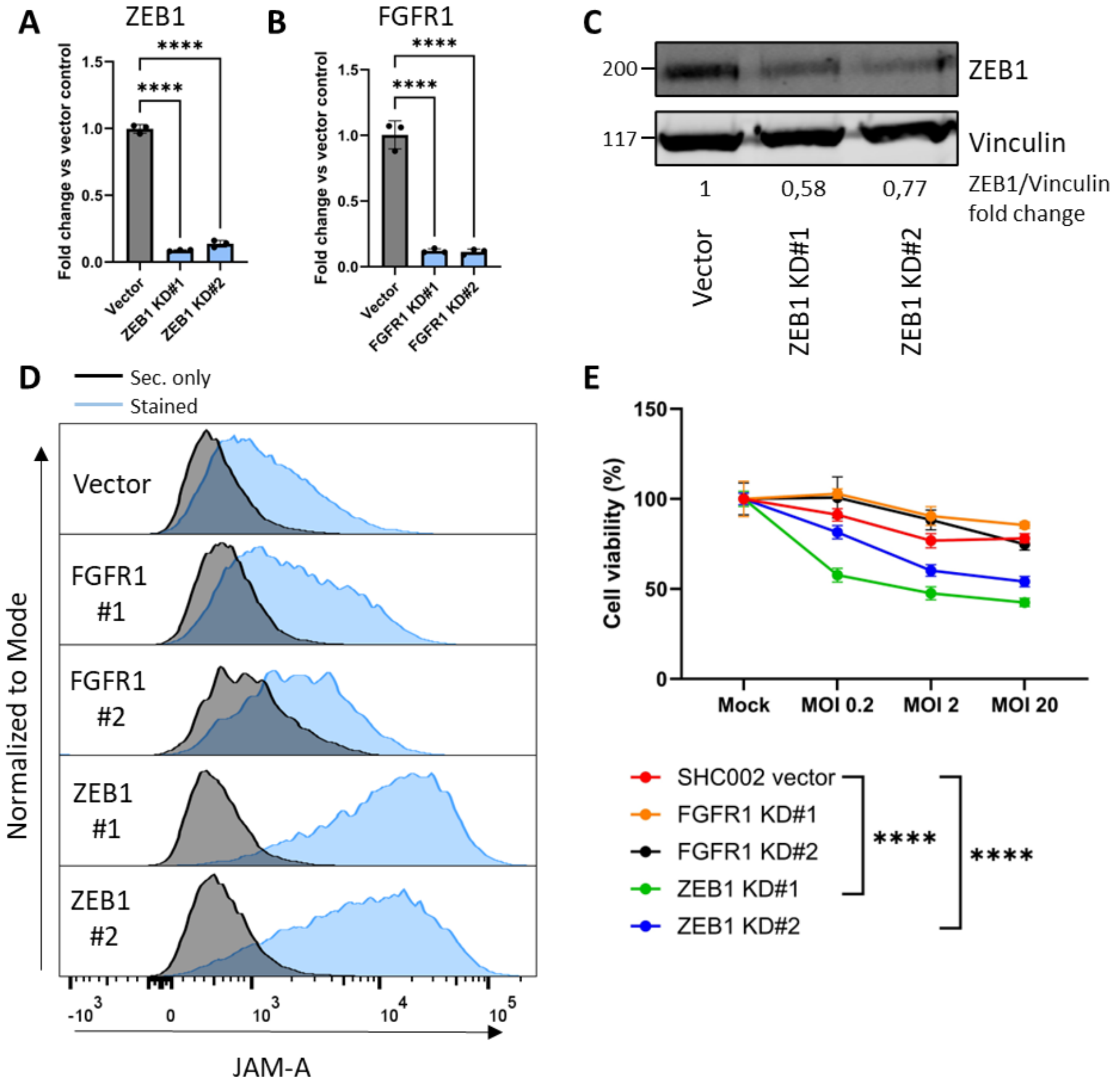

Figure S1. Knockdown of ZEB1, but not FGFR1, in a skin fibroblast cell line NBS results in JAM-A upregulation and sensitization to reovirus-mediated cell death. **A,B** RT-qPCR for *ZEB1* in NBS vector control and ZEB1 KD (A) and *FGFR1* in NBS vector control and FGFR1 KD (B). Ct values were corrected for *β-actin* expression and calculated as fold change vs vector control. ****p≤0,0001 as determined by one-way ANOVA with correction for multiple testing (Šídák's test). Data is derived from a representative experiment and plotted as mean ± SD. **C** Western blot for ZEB1 (200 kDa) with vinculin (117 kDa) as loading control. Band intensity was determined using Image Studio Lite software and calculated as fold change versus the WT cell line. **D** Flow cytometric analysis of cell-surface JAM-A expression in NBS vector

control and FGFR1 and ZEB1 KD. Black: secondary antibody only, blue: stained. **D** Cell viability (%) relative to mock following infection with reovirus at multiple MOIs for 5 days, as measured by a WST-1 assay. Significance was calculated using two-way ANOVA with correction for multiple testing (Šídák's test), significance is depicted at MOI 10, ****p≤0,0001. Data is derived from a representative experiment and plotted as mean ± SD.

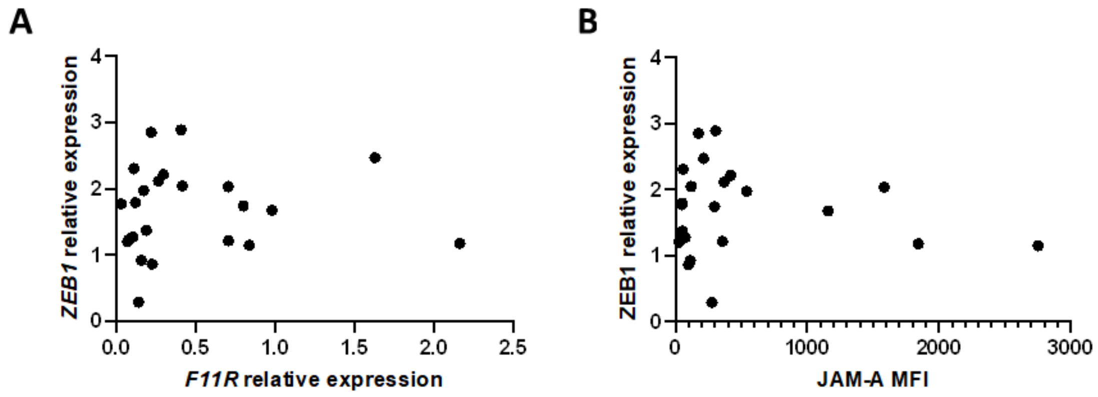

Figure S2. ZEB1 is expressed in a panel of patient-derived CAFs, but does not correlate to JAM-A expression. **A** RT-qPCR for *ZEB1* and *F11R* in 23 patient-derived CAFs (12 pancreatic, 3 esophageal, 3 gastric and 5 duodenal CAFs), described in Harryvan *et al.* Cancer Gene Ther. (2022). Ct values were corrected for *IPO8* and *EIF2B1* expression. **B** qPCR results for *ZEB1* as depicted in A, plotted against JAM-A protein expression (MFI) as published in Harryvan *et al.* Cancer Gene Ther. (2022).

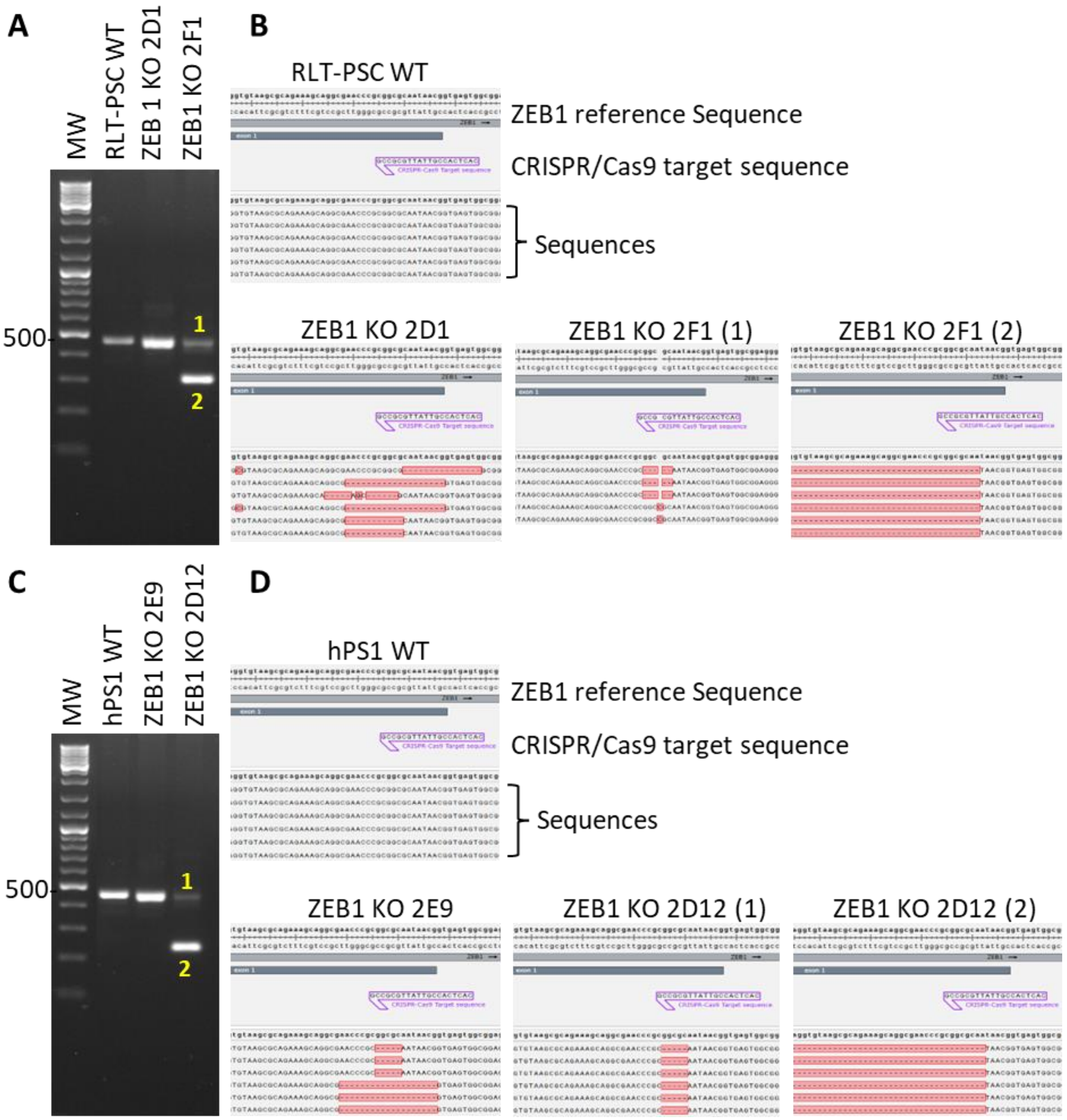

Figure S3. Sequencing of *ZEB1* in the region targeted by the gRNA shows KO-responsible indels. **A** Agarose gel electrophoresis following PCR of RLT-PSC WT and ZEB1 KO clones 2D1 and 2F1. **B** Sanger sequence results of RLT-PSC WT and ZEB1 KO 2D1 and 2F1 (2 different bands) following isolation from the agarose gel, ligation into a vector and sanger sequencing. The different sequences shown are the result of the different colonies isolated from the miniprep. **C** Agarose gel electrophoresis following PCR of hPS1 WT and ZEB1 KO clones 2E9 and 2D12. **D** Sanger sequence results of hPS1 WT and ZEB1 KO 2E9 and 2D12 (2 different bands) following isolation from the agarose gel, ligation into a vector and sanger sequencing. The different sequences shown are the result of the different colonies isolated from the miniprep.

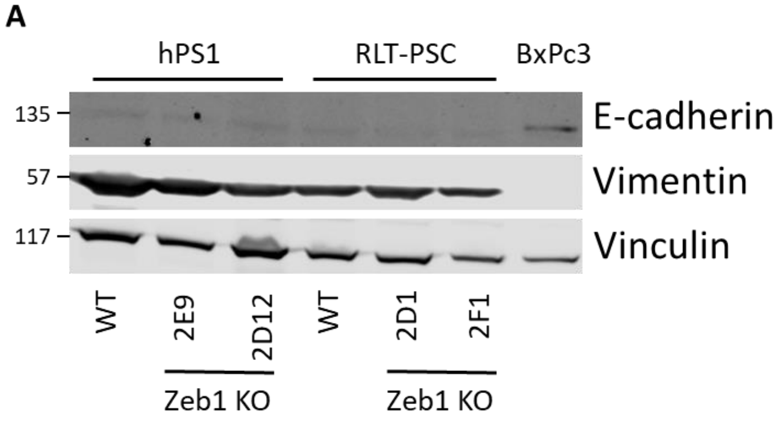

Figure S4. Markers of epithelial-to-mesenchymal transition do not change upon ZEB1 KO in fibroblasts. **A** Western blot for E-cadherin (135 kDa) and vimentin (57 kDa) with vinculin (117 kDa) as loading control.

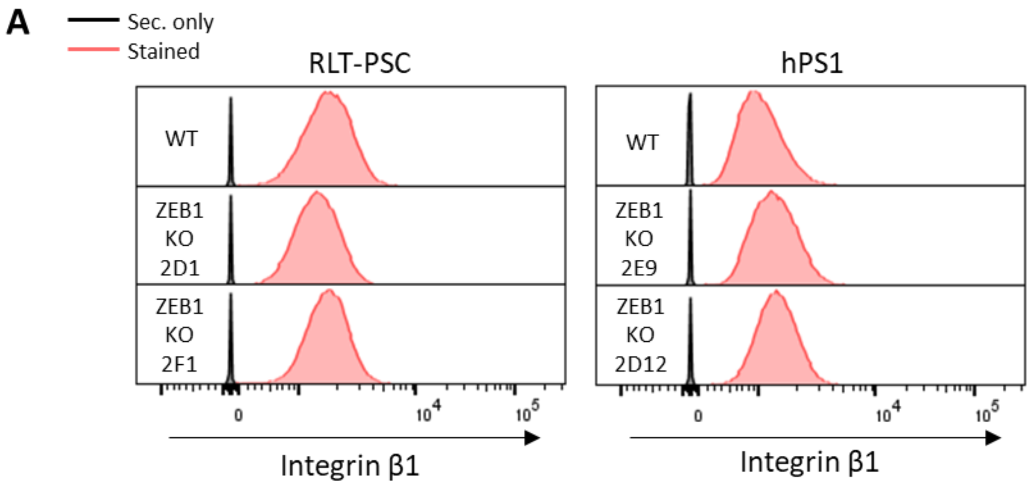

Figure S5. Integrin β1 expression is unchanged following ZEB1 KO. **A** Flow cytometric analyses of Integrin β1 expression in RLT-PSC WT and ZEB1 KO (left panel) and hPS1 WT and ZEB1 KO (right panel). Black: secondary antibody only, red: stained.

Supplemental video legend

Video S1. CellEvent Caspase 3/7 assay in RLT-PSC, hPS1 and KPC3-CAF1 WT and ZEB1 KO following infection with reovirus R124 MOI 10 for 36, 48 and 24 hours, respectively. Phase-contrast and GFP images are overlayed and made into videos using Fiji version 2.14.0. Scale bar: 200 µM.

Supplemental tables

Table S1. Sequences used during the genome-wide CRISPR/Cas9 KO screen

Name	Sequence
Oligo pool	CTTGTGGAAAGGACGAAACACCGNNNNNNNNNNNNNNNNNNNNGTTTAAGAGCTAGAAATAGCAAGTTTAAATAAGGCT
ds_Ultramer	TTTGTCTCAAGATCTAGTTACGCCAAGCTTNNNNNNNNNNGTGACTGGAGTTCAGACGTGTGCTCTTCCGATCAAAAAAGCACCGACTCGGTGCCACTTTTTCAAGTTGATAACGGACTAGCCTTATTTAAACTTGCTATTTCTAGCTC
ds_fw	GGCTTTATATATCTTGTGGAAAGGACGAAACACCG
ds_rev	TTTGTCTCAAGATCTAGTTACGCCAAGC
Final insert	GGCTTTATATATCTTGTGGAAAGGACGAAACACCGNNNNNNNNNNNNNNNNNNNNGTTTAAGAGCTAGAAATAGCAAGTTTAAATAAGGCTAGTCCGTTATCAACTTGAAAAAGTGGCACCGAGTCGGTGCTTTTTTGATCGGAAGAGCACACGTCTGAACTCCAGTCACNNNNNNNNNNAAGCTTGGCGTAACTAGATCTTGAGACAAA
PCR1_fw	GGACTATCATATGCTTACCGTAACTTGAAAGTATTTCG
PCR1_rev	CTTTAGTTTGTATGTCTGTTGCTATTATGTCTACTATTCTTTCC
PCR2_fw	ACACTCTTTCCCTACACGACGCTCTTCCGATCTCTTGTGGAAAGGACGAAACAC
PCR2_rev	AGAAGACGGCATACGAGATCTGCCATTTGTCTCAAGATCTAGTTAC
PCR3_fw	AATGATACGGCGACCACCGAGATCTACAC[I5]ACACTCTTTCCCTACACGACGCTCT
PCR3_rev	CAAGCAGAAGACGGCATACGAGATCTGCCATTTG
CRISPRSeq	CGATCTCTTGTGGAAAGGACGAAACACCG

Table S2. Mission shRNA constructs

**Mission shRNA constructs**	**Sequence (5'-3')**
Non-targeting control SHC002	CCGGCAACAAGATGAAGAGCACCAACTCG-<br>AGTTGGTGCTCTTCATCTTGTTGTTTTT
Mouse ZEB1 KD#1 (TRCN0000235850)	ATAGAGGCTACAAGCGCTTTA
Mouse ZEB1 KD#2 (TRCN0000235853)	GTCGACAGTCAGTAGCGTTTA
Mouse FGFR1 KD#1 (TRCN0000023295)	CCTGGAGCATCATAATGGATT
Human ZEB1 KD#1 (TRCN0000017565)	CCTCTCTGAAAGAACACATTA
Human ZEB1 KD#2 (TRCN0000364631)	CCTACCACTGGATGTAGTAAA
Human FGFR1 KD#1 (TRCN0000312574)	TGCCACCTGGAGCATCATAAT
Human FGFR1 KD#2 (TRCN0000121185)	CCACAGAATTGGAGGCTACAA
Human SPPL3 KD#1 (TRCN0000307051)	CCTGGTCTCCTACTATGCTTT
Human SPPL3 KD#2 (TRCN0000308107)	GGGCATCGGAGACATCGTTAT

Table S3. Primer sequences

Gene	Primer sequence (5'-3')
M13 Reverse sequencing primer	CAGGAAACAGCTATGAC
ZEB1 KO validation	Fw: CACCACACCTGAGGAAAAC<br>Rv: TTTCCCACTCCACTTTGCCGTC
ChIP *F11R* 1	Fw: GCCTGCAACATCTCCCGTT<br>Rv: ATGTTAAGGGCTTCTGCGGTG
ChIP *F11R* 2	Fw: ACAGGAGCTGCCTCAGATTGG<br>Rv: GTACTTCTCAGCCCTCTAGCTC
ChIP *F11R* 3	Fw: ACTACAGCGAGGGGACTGAG<br>Rv: GAAGAGCAGCGGTTCCTTAC
ChIP *F11R* 4	Fw: TGCACGTTCCGATTGGTGTAA<br>Rv: AGTCTCCTGGGCCAATCTGAG
ChIP *E-Cadherin*	Fw: GGCCGGCAGGTGAAC<br>Rv: GGGCTGGAGTCTGAACTGAC
ACTB (mouse)	Fw: AGGTCATCACTATTGGCAACGA<br>Rv: CCAAGAAGGAAGGCTGGAAAA
MZT22 (mouse)	Fw: TCGGTGCCCATATCTCTGTC<br>Rv: CTGCTTCGGGAGTTGCTTTT
PTP4A2 (mouse)	Fw: AGCCCCTGTGGAGATCTCTT<br>Rv: AGCATCACAAACTCGAACCA
ZEB1 (mouse)	Fw: ATTCAGCTACTGTGAGCCCTGC<br>Rv: CATTCTGGTCCTCCACAGTGGA
FGFR1 (mouse)	Fw: GCCTCACATTCAGTGGCTGAAG<br>Rv: AGCACCTCCATTTCCTTGTCGG
F11R (mouse)	Fw: CACCTACTCTGGCTTCTCCTCT<br>Rv: TGCCACTGGATGAGAAGGTGAC
IPO8 (human)	Fw: AGGATCAGAGGACAGCACTGCA

	Rv: AGGTGAAGCCTCCCTGTTGTTC
EIF2B1 (human)	Fw: CTACTCCAGAGTGGTCCTGAGA<br>Rv: GTTGAGGTGGCAGAGGGCTTTG
F11R (human)	Fw: GTGAAGTTGTCCTGTGCCTACTC<br>Rv: ACCAGTTGGCAAGAAGGTCACC
ZEB1 (human)	Fw: GGCATACACCTACTCAACTACGG<br>Rv: TGGGCGGTGTAGAATCAGAGTC
FGFR1 (human)	Fw: GCACATCCAGTGGCTAAAGCAC<br>Rv: AGCACCTCCATCTCTTTGTCGG

Table S4. List of genes replicate 1 CRISPR/Cas9 screen

Table S5. List of genes replicate 2 CRISPR/Cas9 screen
